# Attitudes of Chinese residents toward sugar-sweetened beverage tax and their willingness to pay: a cross-sectional survey

**DOI:** 10.3389/fnut.2023.1268436

**Published:** 2023-10-25

**Authors:** Xuanfei Zhang, Jinrui Bai, Mengyao Xian, Junmao Sun, Haiquan Xu

**Affiliations:** ^1^Institute of Food and Nutrition Development, Ministry of Agriculture and Rural Affairs, Beijing, China; ^2^Business School, Beijing Normal University, Beijing, China; ^3^Chinese Academy of Agricultural Sciences, Beijing, China

**Keywords:** sugar-sweetened beverages, sugar-sweetened beverage tax, tax attitude, influencing factors, willingness to pay

## Abstract

**Background:**

Excessive consumption of sugar-sweetened beverages (SSBs) is associated with increased risks of obesity and chronic diseases. To effectively control SSB consumption, several countries including Mexico, France, and the United Kingdom have implemented SSB taxes. However, research on SSB taxes in China is limited.

**Objective:**

To assess the attitudes of Chinese residents toward the SSB tax and their willingness to pay the tax. Methods: Data were collected through a questionnaire survey among 881 respondents. The generalized ordered logit regression model and marginal effect analysis were used to analyze Chinese participants’ attitudes toward SSB tax and their willingness to pay it.

**Results:**

The average monthly expenditure on SSBs was 44.8 ± 45.3 Yuan (RMB) (6.95 ± 7.02$), and 54.6% of residents supported the SSB tax; they were willing to pay, on average, 1.19 times the original price after additional tax. Age, physical exercise, self-rated health status, weight control plan, awareness of SSBs, children’s consumption of SSBs, and proximity to the nearest SSB outlet significantly influenced attitudes toward tax. Notably, SSB awareness had the greatest effect on tax attitudes, with a 17% increase in the probability of supporting SSB tax for every one-level increase in SSB awareness among residents.

**Conclusion:**

Residents in China have attained a certain level of awareness of, support for, and willingness to pay SSB tax. However, promoting knowledge about the health effects of SSBs and conducting further research to evaluate the effect of SSB tax on obesity prevention in China is still essential.

## 1. Introduction

Currently, the consumption of sugar-sweetened beverages is on the rise in various countries (1). In China, the rising beverage consumption led to a significant increase in beverage production, with sugar-sweetened beverages (SSBs) accounting for more than half of the Chinese beverage market (2). However, excessive SSB consumption is associated with increased risks of obesity, type 2 diabetes, cardiovascular disease, and other chronic diseases (3–5). Furthermore, SSB intake is linked to a decrease in calcium and other nutrient intake from milk (6). To address the problem of excessive SSB consumption, several countries including Mexico, France, and the United States have implemented SSB taxes since 2011. In France, a 3.3% decrease in soft drink sales was reported within 4 months of implementing taxes on drinks based on sugar content in 2012 (7). Similarly, in Mexico, a 12% reduction in SSB consumption occurred 1 year after implementing a tax of 1 peso/L on SSBs in 2014 (8). In Berkeley, USA, SSB consumption declined by 9.6% following the introduction of a 1¢/oz. tax on SSBs in 2015 (6). In 2016, the World Health Organization (WHO) recommended a 20% tax rate on SSBs for obesity prevention in member states (9). Although some studies have explored SSB taxation in China (10, 11), most of them have been qualitative and have focused on reviewing or analyzing foreign tax systems (10–15). The empirical research is rare. Only Chong et al. investigated Hong Kong residents’ willingness to pay the SSB tax and revealed week support among the residents, with only 60% of them supporting tax rates of 5–10% (16). The present study assessed the attitudes of Chinese residents toward the SSB tax and their willingness to pay this tax. Moreover, the factors influencing the residents’ attitudes toward the SSB tax were analyzed to provide a theoretical basis for SSB tax policy development in China.

## 2. Materials and methods

### 2.1. Data collection

A web-based survey was conducted on the Credamo data research platform, which is a one-stop professional survey and experiment tool online. Before the formal survey, three pilot studies were implemented to revise and refine the questionnaire and determine the required sample size. The three pilot studies were conducted among 60, 34, and 67 participants respectively, totaling 161 participants with 60 Credamo users online and 101 participants in person. The results of the pilot surveys indicated the high reliability and validity of the questionnaire, with Cronbach’s coefficient and a Kaiser–Meyer–Olkin value of 0.807 and 0.819, respectively. By combining the pilot data and using a sample size formula for estimating the overall mean, the sample size was estimated to be 800. The participants were recruited through the Credamo data research platform and the questionnaire was mainly distributed online in 2021, with participants spanning across the provinces of Chinese mainland. The formal questionnaire consisted of 29 questions, and the pilot studies showed that it took about 5 min to complete the questionnaire. The questionnaire comprises three main sections: general characteristics information (such as gender, age, height, weight, education level, income, exercise habits, and self-rated health), expenditure on SSB consumption, and attitudes toward SSB taxes (including beverage awareness, attitudes toward taxation, and willingness to pay taxes). Invalid questionnaires were identified and excluded using specific questions. Ultimately, a total of 1,121 questionnaires were collected online, and after exclusion of invalid questionnaires, 881 valid questionnaires were used for further analysis, resulting in an effective recovery rate of 78.59%.

### 2.2. Variables

To assess residents’ attitudes toward SSB consumption and SSB tax, two questions were used, which were rated on a 5-point Likert scale: “Do you think frequent SSB consumption is harmful to health?” and “Do you support additional tax on SSBs?” The residents’ willingness to pay the SSB tax was measured by asking, “How much would you be willing to pay for a bottle of 500-mL beverage with an original price of 3.5 RMB (0.54$) if taxes were included?” Overweight and obesity were defined according to the People’s Republic of China Health Standards-Adult Weight Determination (17). Frequent SSB consumption was defined as consuming SSBs ≥4 times a week (18). The annual *per capita* household income was categorized into low-income [< 20,000 RMB (3,093$)], middle-low-income [20,000–50,000 RMB (3,093–7,733$)], middle-income [50,000–80,000 RMB (7,733–12,372$)], middle-high-income [80,000–110,000 RMB (12,372.00-17,0112$)], and high-income [> 110,000 RMB (17,0112$)] groups according to the order from low to high. Different regions were classified as eastern, central, and western according to the National Bureau of Statistics (19).

### 2.3. Statistical analysis

The logit regression model was employed to analyze the factors influencing Chinese residents’ attitudes toward the SSB tax. The residents’ attitude toward the SSB tax (W) was considered an ordered discrete variable, with values ranging from 1 to 5. The generalized ordered logit model was used in this study (20), which allows the relaxation of the parallel line assumption and captures the order information of dependent variables. The greater value of positive model regression coefficients in the model indicate a higher likelihood of residents supporting the SSB tax, and the greater value of the negative coefficients indicate a higher likelihood of residents opposing the SSB tax. The explanatory variables were classified into three types: socioeconomic characteristics, including sex, age, income, education level, residence, student status (be student or not), and whether their major or job was food nutrition-related; health status, including body mass index (BMI), physical exercise frequency, self-assessed health status, and intention or behavior to lose weight in the past year; and SSB consumption, including average monthly SSB consumption expenditure, attitude toward regular consumption of SSBs, frequency of SSB consumption among children and adults in the household, and distance to the nearest SSB outlet. Model fitting was performed using the maximum likelihood ratio test.

### 2.4. Marginal effect analysis

The estimation of coefficients in the generalized ordered logit regression model only provides information on the statistical significance and direction of the action of the variables. To gain a specific understanding of the effect of each variable on the dependent variable, the marginal effects must be calculated. The marginal effect represents the effect of a change in the independent variable on the probability of an individual selecting a specific category, with other factors maintained constant (21). The average marginal effects of each explanatory variable were calculated; this value represents the average of the marginal effects across different values of the explanatory variable. Statistical analysis was performed using Stata 15.0.

## 3. Results

### 3.1. Participant characteristics

Among 881 respondents, 345 (39.2%) were men, and the majority of the respondents (56.6%) were from the eastern region. In terms of age distribution, the largest proportions were observed in the 21–25 years (39.3%) and 26–30 years (25.5%) groups. Urban residents accounted for 89% of the participants. Regarding the education level, 85.8% of the respondents had completed university education or higher. In terms of the annual household income, the majority of the study population had an annual household income between 20,000 and 80,000 RMB (3,093–12,372$) *per capita* (Table 1).

**Table 1:** Sociodemographic characteristics and nutritional status of Chinese adults evaluated in 2021 (*n* = 881).

Variable	Classification	*n* (%)
Total		881 (100.0)
Sex	Male	345 (39.2)
	Female	536 (60.8)
Age	≤20	79 (9.0)
	21 ~ 25	346 (39.3)
	26 ~ 30	225 (25.5)
	31 ~ 35	140 (15.9)
	≥36	91 (10.3)
Nutritional status	Overweight or obesity	160 (18.2)
	Other	721 (81.8)
Education level	High School and below	35 (4.0)
	Specialties	90 (10.2)
	Undergraduate	627 (71.2)
	Master’s degree and above	129 (14.6)
Students	Yes	379 (43.0)
	No	502 (57.0)
career related to Food Nutrition	Yes	115 (13.1)
	No	766 (87.0)
Annual household income (*per capita*, RMB)	≤20,000 (3,093$)	87 (9.9)
	20,000 ~ 50,000 (3,093–7,733$)	252 (28.6)
	50,000 ~ 80,000 (7,733–12,372$)	260 (29.5)
	80,000 ~ 110,000 (12,372–17,012$)	133 (15.1)
	≥110,000 (17,012$)	149 (16.9)
Residence	Urban	784 (89.0)
	Rural	97 (11.0)
Area	Eastern	497 (56.5)
	Central	235 (26.7)
	Western	147 (16.7)

*n*, the number of participants; %, percentage. The exchange rate conversion time was recorded as 2021, UTC.

### 3.2. Expenditure, perceptions, and attitudes toward SSB tax

#### 3.2.1. Expenditure on SSBs

The average monthly expenditure on SSBs was 44.8 ± 45.3 Yuan (RMB) (6.95 ± 7.02$), with a range of 0 (6.8% of participants) to 300 Yuan (0 to 46.39$). Notably, male residents had a significantly higher average expenditure on SSBs compared with female residents, with a difference of 8.98 Yuan (1.39$) (*p* < 0.01). Furthermore, overweight individuals (or obese individuals) had a higher average expenditure on SSBs [45.75 ± 46.03 Yuan ($7.09 ± 7.12)] than those who were not overweight [40.68 ± 41.65 Yuan (6.30 ± 6.45$)]. Urban residents had a slightly higher expenditure on SSBs than rural residents. Age, education level, and income level were found to play crucial roles in SSB expenditure (Table 2).

**Table 2:** Chinese adults’ expenditure on SSB according sociodemographic characteristics and nutritional status, 2021 (*n* = 881).

Variable	Classification	SSB expenditures/month (Mean ± SD)	*F*
Total		44.80 ± 45.30 Yuan (6.95 ± 7.02$)	
Sex	Male	50.29 ± 49.04 Yuan (7.79 ± 7.60$)	
	Female	41.32 ± 42.37 Yuan (6.40 ± 6.56$)	8.31**
Residence	Urban	45.46 ± 45.54 Yuan (7.05 ± 7.07$)	
	Rural	39.78 ± 43.05 Yuan (6.17 ± 6.67$)	1.36
Age	≤20	25.53 ± 28.79 Yuan (3.96 ± 4.47$)	
	21 ~ 25	34.29 ± 34.57 Yuan (5.31 ± 5.36$)	
	26 ~ 30	55.39 ± 48.54 Yuan (8.59 ± 7.53$)	
	31 ~ 35	63.27 ± 55.78 Yuan (9.80 ± 8.63$)	
	≥36	47.19 ± 50.16 Yuan (7.32 ± 7.77$)	18.56**
Weight status	Overweight and obese	45.75 ± 46.03 Yuan (7.09 ± 7.12$)	
	Other	40.68 ± 41.65 Yuan (6.30 ± 6.45$)	1.65
Education level	High School and below	40.83 ± 36.34 Yuan (6.32 ± 5.62$)	
	Specialized	49.59 ± 44.42 Yuan (7.68 ± 6.88$)	
	Undergraduate	46.44 ± 47.18 Yuan (7.19 ± 7.31$)	
	Master’s degree or above	34.78 ± 36.81 Yuan (5.39 ± 5.70$)	2.82*
Income level	≤20,000 RMB	21.67 ± 19.13 Yuan (3.36 ± 2.97$)	
	20,000 ~ 50,000RMB	35.81 ± 36.33 Yuan (5.55 ± 5.63$)	
	50,000 ~ 80,000 RMB	45.65 ± 42.67 Yuan (7.08 ± 6.61$)	
	80,000 ~ 110,000RMB	56.71 ± 48.88 Yuan (8.80 ± 7.58$)	
	≥110,000RMB	61.58 ± 59.68 Yuan (9.55 ± 9.25$)	16.70**

*, **Indicate significant differences at the 5, and 1% levels, respectively. SSB, sugar-sweetened beverage; F, F-statistics. The exchange rate conversion time was recorded as 2021, UTC.

#### 3.2.2. Attitude on the health effects of SSBs

Among the residents surveyed, 53.1% believed that the regular consumption of SSBs influenced health, whereas 30.1% believed that it had a high influence. By contrast, only 0.57% believed that SSB consumption had no influence on health. Residents with a higher expenditure on SSBs were more likely to perceive that regular SSB consumption influences health. Significant differences were observed between sex and age groups. A greater proportion of women and older residents believed that regular SSB consumption had a greater impact on health compared with the men and younger counterparts (Table 3).

**Table 3:** Chinese adults’ attitudes toward the effects of regular SSB consumption on health according sociodemographic characteristics and nutritional status, 2021 (*n* = 881).

Variable	Classification	No impact [*n* (%)]	Low impact [*n* (%)]	Moderate impact [n (%)]	High Impact [*n* (%)]	Higher impact [*n* (%)]	χ^2^
Total							
Sex	Male	1 (0.29)	17 (4.93)	59 (17.10)	169 (48.99)	99 (28.70)	16.236**
	Female	4 (0.75)	21 (3.92)	46 (8.58)	299 (55.78)	166 (30.97)	
Age	≤20	1 (1.27)	2 (2.53)	10 (12.66)	44 (55.70)	22 (27.85)	31.345*
	21 ~ 25	3 (0.87)	19 (5.49)	40 (11.56)	182 (52.60)	102 (29.48)	
	26 ~ 30	0 (0.00)	11 (4.89)	31 (13.78)	132 (58.67)	51 (22.67)	
	31 ~ 35	0 (0.00)	5 (3.57)	20 (14.29)	69 (49.29)	46 (32.86)	
	≥36	1 (1.10)	1 (1.10)	4 (4.40)	41 (45.05)	44 (48.35)	
Nutritional status	Overweight or obesity	1 (0.63)	3 (1.88)	17 (10.63)	88 (55.00)	51 (31.88)	3.320
	Other	4 (0.55)	35 (4.85)	88 (12.21)	380 (52.70)	214 (29.68)	
Education level	High School and below	0 (0.00)	1 (2.86)	3 (8.57)	16 (45.71)	15 (42.86)	11.093
	Specialized	0 (0.00)	2 (2.22)	12 (13.33)	47 (52.22)	29 (32.22)	
	Undergraduate	4 (0.64)	33 (5.26)	76 (12.12)	339 (54.07)	175 (27.91)	
	Master’s degree or above	1 (0.78)	2 (1.55)	14 (10.85)	66 (51.16)	46 (35.66)	
Residence	Urban	0 (0.00)	4 (4.12)	15 (15.46)	52 (53.61)	26 (26.80)	2.171
	Rural	5 (0.64)	34 (4.34)	90 (11.48)	416 (53.06)	239 (30.48)	
Income level	≤20,000 RMB	0 (0.00)	8 (9.20)	7 (8.05)	46 (52.87)	26 (29.89)	16.329
	20,000 ~ 50,000RMB	2 (0.79)	5 (1.98)	29 (11.51)	138 (54.76)	78 (30.95)	
	50,000 ~ 80,000 RMB	2 (0.77)	12 (4.62)	31 (11.92)	147 (56.54)	68 (26.15)	
	80,000 ~ 110,000RMB	0 (0.00)	5 (3.76)	17 (12.78)	68 (51.13)	43 (32.33)	
	≥110,000RMB	1 (0.67)	8 (5.37)	21 (14.09)	69 (46.31)	50 (33.56)	

*, **Indicate significant differences at the 5, and 1% levels, respectively. SSB, sugar-sweetened beverage; n, the number of participants; %, percentage; χ^2^, χ^2^statistics.

#### 3.2.3. Attitude on SSB tax

This study revealed that 54.6% of the participants supported or strongly supported the implementation of the SSB tax (16.1 and 38.5%, respectively). However, 6.0, 17.7, and 21.7% of the participants strongly disagreed, somewhat disagreed, and held neutral attitudes, respectively, in this regard. Significant differences were observed in the perceptions of the SSB tax among age groups and household income groups, with older residents and those with higher incomes being more supportive of the SSB tax compared with their counterparts (Table 4). Regarding the residents’ attitudes toward the SSB tax, the following groups were more supportive than their counterparts: older residents, residents who exercised more often, those who perceived themselves to be in better health, those with weight control plans, or those living with children who regularly consume SSBs. Furthermore, the respondents who lived further away from retail outlets were more likely to believed that SSBs had a greater impact on health and support the taxation of SSBs (Supplementary Tables S1, S2). Regarding the use of SSB tax revenues, the top three initiatives supported by respondents were child malnutrition (41.2%), child obesity (30.8%), and health education (14.7%).

**Table 4:** Chinese adults’ attitudes toward SSB tax according sociodemographic characteristics and nutritional status, 2021 (*n* = 881).

Variable	Classification	Strongly nonsupport [*n* (%)]	Nonsupport [*n* (%)]	Neutral [*n* (%)]	Support [*n* (%)]	Strongly support [*n* (%)]	χ^2^
Total							
Sex	Male	27 (5.18)	99 (19.00)	112 (21.5)	204 (39.16)	79 (15.16)	2.849
	Female	21 (6.38)	52 (15.81)	66 (20.06)	131 (39.82)	59 (17.93)	
Age	≤20	7 (8.97)	17 (21.79)	19 (24.36)	26 (33.33)	9 (11.54)	70.308**
	21 ~ 25	20 (6.06)	78 (23.64)	79 (23.94)	117 (35.45)	36 (10.91)	
	26 ~ 30	9 (4.13)	40 (18.35)	46 (21.1)	95 (43.58)	28 (12.84)	
	31 ~ 35	4 (2.96)	12 (8.89)	20 (14.81)	63 (46.67)	36 (26.67)	
	≥36	8 (8.99)	4 (4.49)	14 (15.73)	34 (38.2)	29 (32.58)	
Nutritional status	Overweight and obese	13 (8.72)	17 (11.41)	30 (20.13)	63 (42.28)	26 (17.45)	
	Other	35 (4.99)	134 (19.12)	148 (21.11)	272 (38.8)	112 (15.98)	7.736
Education level	High School and below	3 (9.38)	6 (18.75)	6 (18.75)	10 (31.25)	7 (21.88)	13.196
	Specialized	7 (7.95)	12 (13.64)	22 (25)	29 (32.95)	18 (20.45)	
	Undergraduate	28 (4.55)	108 (17.53)	132 (21.43)	250 (40.58)	98 (15.91)	
	Master’s degree or above	10 (8.77)	25 (21.93)	18 (15.79)	46 (40.35)	15 (13.16)	
Residence	Urban	4 (4.17)	22 (22.92)	15 (15.63)	41 (42.71)	14 (14.58)	3.978
	Rural	44 (5.84)	129 (17.11)	163 (21.62)	294 (38.99)	124 (16.45)	
Income level	≤20,000 RMB	6 (6.9)	14 (16.09)	19 (21.84)	36 (41.38)	12 (13.79)	26.644*
	20,000 ~ 50,000RMB	10 (4.18)	51 (21.34)	60 (25.1)	90 (37.66)	28 (11.72)	
	50,000 ~ 80,000 RMB	18 (7.17)	40 (15.94)	49 (19.52)	105 (41.83)	39 (15.54)	
	80,000 ~ 110,000RMB	9 (7.03)	25 (19.53)	20 (15.63)	54 (42.19)	20 (15.63)	
	≥110,000RMB	5 (3.45)	21 (14.48)	30 (20.69)	50 (34.48)	39 (26.9)	

*, **Indicate significant differences at the 5, and 1% levels, respectively. SSB, sugar-sweetened beverage; n, the number of participants; %, percentage; χ^2^, χ^2^statistics.

#### 3.2.4. SSB-purchasing behavior

Following the hypothetical implementation of a 20% SSB tax, 95.9% of the respondents indicated that their SSB purchase behavior would change. Among the respondents, 47.0% stated that they would significantly reduce their SSB purchases, 23.4% stated that they would reduce their purchases by half, 17.5% stated that they would slightly reduce their purchases, and 4.1% stated that they would completely stop purchasing SSBs. As SSB consumption decreased, the consumption of alternative beverages increased. The alternatives to SSBs that could be selected by the respondents included milk tea drinks, milk and yogurt, 100% fruit and vegetable juices, mineral water, sugar-free or low-sugar beverages, desserts and candies, and fruits, among which the top three choices were milk and yogurt (47.5%), water (17.4%), and sugar-free beverages (13.5%).

#### 3.2.5. Willingness to pay SSB tax

The proportions of the respondents who were willing to pay more than 5.0, 4.5, and 4.0 Yuan (0.77, 0.70, and 0.62$) for a 500-mL bottle of beverage with an original price of 3.50 Yuan (0.54$) were 14.51, 20.58, 81.52%, respectively. The respondents were willing to pay 4.16 Yuan (0.64$) on average. If the tax is fully passed on to consumers, the tax rate would be approximately 19% based on the price increase from 3.50 Yuan to 4.16 Yuan. As the tax rate increases, some consumers may reduce their consumption of SSBs. Furthermore, the attitude toward the SSB tax was significantly positively correlated with the maximum willingness to pay the tax (Table 5).

**Table 5:** Average willingness of Chinese adults with sociodemographic characteristics and nutritional status to pay for SSB, 2021 (*n* = 881).

Variable	Classification	Payment (Mean ± SD)	Tax rate	*F*
Sex	Male	4.18 ± 0.73 Yuan (0.65 ± 0.11$)	19%	0.09
	Female	4.16 ± 0.64 Yuan (0.64 ± 0.10$)	19%	
Age	≤20	4.19 ± 0.47 Yuan (0.65 ± 0.07$)	20%	0.71
	21 ~ 25	4.19 ± 0.84 Yuan (0.65 ± 0.13$)	20%	
	26 ~ 30	4.12 ± 0.40 Yuan (0.64 ± 0.06$)	18%	
	31 ~ 35	4.26 ± 0.86 Yuan (0.66 ± 0.13$)	22%	
	≥36	4.04 ± 0.41 Yuan (0.63 ± 0.06$)	15%	
Nutritional status	Overweight and obese	4.09 ± 0.47 Yuan (0.63 ± 0.07$)	17%	1.29
	Other	4.19 ± 0.74 Yuan (0.65 ± 0.10$)	20%	
Education level	High School and below	3.99 ± 0.43 Yuan (0.62 ± 0.07$)	14%	0.21
	Specialized	4.18 ± 0.83 Yuan (0.65 ± 0.13$)	19%	
	Undergraduate	4.18 ± 0.73 Yuan (0.65 ± 0.11$)	19%	
	Master’s degree or above	4.18 ± 0.47 Yuan (0.65 ± 0.07$)	19%	
Residence	Urban	4.29 ± 1.08 Yuan (0.67 ± 0.17$)	23%	1.17
	Rural	4.16 ± 0.65 Yuan (0.64 ± 0.10$)	19%	
Income level	≤20,000 RMB	4.20 ± 0.43 Yuan (0.65 ± 0.07$)	20%	0.17
	20,000 ~ 50,000RMB	4.18 ± 0.80 Yuan (0.65 ± 0.12$)	19%	
	50,000 ~ 80,000 RMB	4.13 ± 0.69 Yuan (0.64 ± 0.11$)	18%	
	80,000 ~ 110,000RMB	4.21 ± 0.45 Yuan (0.65 ± 0.07$)	20%	
	≥110,000RMB	4.18 ± 0.81 Yuan (0.65 ± 0.13$)	19%	
SSB awareness	No impact	5.00 ± 0 Yuan (0.78 ± 0$)	43%	1.23
	Low impact	3.96 ± 0.38 Yuan (0.61 ± 0.06$)	13%	
	Moderate impact	4.17 ± 0.75 Yuan (0.65 ± 0.12$)	19%	
	Higher impact	4.14 ± 0.58 Yuan (0.64 ± 0.09$)	18%	
	High impact	4.26 ± 0.89 Yuan (0.66 ± 0.14$)	22%	
The attitude toward SSB Tax	Strongly Disagree	3.91 ± 0.31 Yuan (0.60 ± 0.05$)	12%	4.59**
	Somewhat Disagree	4.07 ± 0.60 Yuan (0.63 ± 0.09$)	16%	
	Neutral	4.03 ± 0.38 Yuan (0.62 ± 0.06$)	15%	
	Somewhat Agree	4.21 ± 0.63 Yuan (0.65 ± 0.10$)	20%	
	Strongly Agree	4.45 ± 1.13 Yuan (0.69 ± 0.18$)	27%	

*, **Indicate significant differences at the 5, and 1% levels, respectively. SSB, sugar-sweetened beverage; F, F-statistics. The exchange rate conversion time was recorded as 2021, UTC.

### 3.3. Effects of different factors on attitude toward SSB tax

The marginal effect analysis revealed that for every one-level increase in SSB awareness (the resident’s perception of the health effects of regular SSB consumption), the probability of somewhat disagreeing and being neutral decreased by 6.5 and 5.9%, respectively, whereas the probability of being supportive of the SSB tax increased by 17%. Frequent SSB consumption by adults in the household increased the probability of more opposition of the tax by 6.6% and reduced the probability of providing more supportive of the tax by 9.7%. An increase of one level in the distance to the nearest SSB outlet resulted in a 4.9% increase in the probability of somewhat agreeing. Moreover, it led to a decrease of 4.2% in the probability of somewhat disagreeing and a decrease of 3.6% in the probability of being neutral. Furthermore, a one-level increase in physical exercise frequency resulted in a 4.9% increase in the probability of somewhat agreeing and a 3.9% increase in the probability of strongly agreeing. For self-assessed health status, an increase of one level led to a 4.9% increase in the probability of strongly agreeing. Regarding age, for every 1-year increase, the probability of somewhat disagreeing decreased by 1%, whereas the probability of somewhat agreeing and strongly agreeing increased by 0.6 and 0.3%, respectively. Lastly, the probability of rural residents being neutral was 11.2% higher than that for urban residents (Table 6).

**Table 6:** Marginal effects of explanatory variables under different thresholds for Chinese adults according sociodemographic characteristics and nutritional status, 2021 (*n* = 881).

Variable	W = 1	W = 2	W = 3	W = 4	W = 5
SSB expenses	−0.0002	0.0006	0.0000	0.0007	−0.0010
Expenditures on SSB in quadratic terms	0.0000	0.0000	0.0000	0.0000	0.0000
Perception of SSB	−0.0199	0.0650**	0.0590**	−0.0259	0.1698**
Are there children in the family who regularly consume the SSB	−0.0242	−0.0400	−0.0226	0.0526	0.0342
Are there any adults in the household who regularly consume the SSB	0.0215	0.0656**	0.0092	0.0971**	0.0008
Distance to the nearest SSB outlet	−0.0051	−0.0239	−0.0097	0.0491**	−0.0104
Physical exercise frequency	−0.0096	0.0421**	−0.0361*	0.0493*	0.0385*
Self-assessed health status	−0.0155	−0.0305	−0.0159	0.0134	0.0486**
Whether there is the intention or behavior to lose weight in the recent year	0.0289*	−0.0349	0.0452*	−0.0020	0.0206
Sex	0.0111	0.0002	0.0234	−0.0676	0.0330
Age	0.0025	0.0098**	−0.0020	0.0064*	0.0028
BMI	0.0003	0.0005	−0.0047	0.0063	−0.0023
Education	−0.0020	0.0139	−0.0467*	0.0574*	−0.0227
Household income (Logarithm, *per capita*)	0.0146	−0.0094	−0.0087	−0.0306	0.0341
Residence	0.0062	−0.0569	0.1117**	−0.0317	−0.0293
Are you a student	0.0270	−0.0540	0.0275	0.0124	−0.0129
Is the profession related to food nutrition	0.0010	0.0217	−0.0010	−0.0279	0.0061

*, **Indicate significance at 5, and 1% levels, respectively. SSB, sugar-sweetened beverage; W, the dependent variable (attitudes of residents toward sugar-sweetened beverage tax).

## 4. Discussion

The study results revealed that more than half of the consumers expressed support or strong support for the implementation of SSB taxation. The maximum SSB tax, including additional tax, that they were willing to pay 1.19 times the original price, equivalent to a tax rate of 19%. Moreover, age and income significantly influenced the attitudes toward SSB taxation. Furthermore, older individuals or those with higher income levels demonstrated greater support for the SSB tax compared with their counterparts.

SSB consumption is associated with increased energy intake (5), particularly among children and adolescents. SSB overconsumption has been recognized as a potential contributor to overweight or obesity (5, 8). To reduce SSB consumption, many countries have implemented the SSB tax as a policy intervention. However, in China, the SSB tax has not been implemented thus far (21). This is the first study to examine the attitudes and willingness of Chinese individuals to pay SSB tax.

Previous foreign studies have reported varying levels of support for SSB taxes, ranging from 35 to 50%, with some studies even reporting support levels exceeding over 60%. Additionally, when tax revenues were used for specific purposes such as obesity prevention, community fitness facilities subsidies, or other public health programs, the support proportion increased by 13–26% (22–25). However, a survey conducted among rural Michigan residents in the United States revealed that most individuals expressed resistance, distrust, or indifference to SSB taxes (26). Compared with most foreign studies, the results of this study in China revealed a higher level of support for SSB taxes. This finding indicates that a social cognitive basis already exists for the implementation of SSB taxes in China, and the maximum tax rate acceptable is close to the rate of 20% recommended by the WHO.

The support for the SSB tax is influenced by various factors including age, BMI, and education level (27). The present study revealed that Chinese residents who lived with children who frequently consumed SSBs were more likely to support the tax. However, adults who themselves consumed SSBs frequently were more likely to oppose the tax. The marginal effect analysis also revealed that the probability of being supportive decreased by 9.7% for adults who regularly consumed SSBs. This finding suggests that children play a crucial factor in influencing adults’ attitudes toward SSB taxes. Parents are often more concerned about their children’s health and may be more supportive of tax policies that promote children’s health (28). Conversely, if tax policies negatively affect their consumption behavior, residents are more likely to oppose the tax. Notably, our findings differ from those reported by Michelle et al., who found that participants living with one or more adolescents were less supportive of SSB taxes compared with those without children or adolescents (22). Furthermore, the distance to the nearest SSB outlets had a significant positive effect on support for SSB taxes, as indicated by both the obtained regression coefficients and marginal effect analysis results. This finding suggests that easier access to SSBs increases the probability of the residents opposing the tax. Consequently, reducing the availability of SSBs in the environment, particularly in the building environment, may increase the residents’ support for SSB taxes.

Although the SSB tax has gained more attention as a potential intervention measure to address the increasing trend of obesity, experts have raised concerns regarding its effectiveness for obesity prevention in China at the present time. Additional intervention measures should also be considered for SSB retail control. China’s Healthy Oral Action Plan (2019–2025), issued in 2019, already includes measures to restrict SSB retailing and reduce the supply of SSBs in or around primary and secondary schools as well as kindergartens (29). To further strengthen regulatory efforts, mandatory punitive management measures could be implemented. For example, the Chengdu Education Bureau assesses the hygiene of primary and secondary schools (including kindergartens) by randomly checking the SSBs brought to school by students (30). In addition, vending machines in public places such as subways, shopping malls, children’s playgrounds, and hospitals should supply water and healthy beverages instead of SSBs.

To further address the problem of SSB consumption and promote healthier choices, enhancing nutrition knowledge is essential. Regular SSB overconsumption may lead to various health problems (31). Raising awareness about the negative health impacts of excessive SSB consumption and educating the public about the low nutritional quality and high energy content of SSBs are crucial. These goals can be achieved through various means, including increased public campaigns, particularly targeting primary and middle schools to avoid the habit of frequent SSB consumption among children. Increasing support for SSB taxes requires enhancing residents’ perception of the negative effects of SSBs and creating a public consensus on the need for SSB taxation. Factors such as physical activity level, health status, weight control plans, and perception of SSBs can positively influence attitudes toward SSB taxes. In addition, older individuals tend to prioritize health and are more supportive of SSB taxes; however, a study in Kansas, USA, revealed stronger support from younger age groups (32). In the present study, the marginal effect analysis revealed that a one-level increase in SSB awareness could increase the probability of supporting SSB taxes by 17%. Compared with other factors, increased SSB awareness could greatly promote tax support, which is consistent with the result of a study conducted in South Africa (33). Further research should be conducted on various aspects, including residents’ attitudes, countermeasures by enterprises, tax effects, and tax systems, which would provide a basis for the formulation of relevant policies. However, some specialties should be considered for tax in China, such as the form of tax payment.

This study explores residents’ attitudes and willingness to pay for SSB tax, laying the foundation for research in this field and providing practical evidence for relevant policy development. Nevertheless, this study has also several limitations that should be acknowledged. First, due to limited data availability, this study only obtained cross-sectional data for 2021; thus, changes in SSB consumption behavior over time could not be examined. Because SSBs can be addictive, longitudinal data collected at multiple time points would be more suitable for analyzing consumption patterns, so it’s better to have the longitudinal study for further research. Second, the sample primarily consisted of urban young individuals with higher levels of education, potentially resulting in sample bias and limiting the generalizability of the findings to the entire population. Third, the data collected in this study relied on self-reports and assumptions about the implementation of SSB taxes, rather than actual observations of behavior under fiscal measures. This may introduce social desirability bias.

## 5. Conclusion

This study revealed that support for the SSB tax among Chinese residents has reached a certain level, with a willingness to pay a tax rate of 19%. Several factors including age, physical activity, self-rated health status, weight loss plans, and awareness regarding the negative health effects of SSBs were found to influence attitudes toward SSB taxation. Age and income level significantly influenced attitudes toward taxation. Among these factors, the SSB perception had the greatest impact on attitudes toward the SSB tax, with every one-level increase in SSB perception increasing the probability of supporting the SSB tax by 17%. In conclusion, residents in China demonstrated awareness, support, and willingness to pay the SSB tax, indicating a favorable environment for implementing such a tax. However, it is still necessary to continue raising awareness about the health implications of SSB consumption and to conduct further research to assess the potential effects of SSB taxation on obesity prevention in China.

## Data availability statement

The original contributions presented in the study are included in the article/Supplementary material, further inquiries can be directed to the corresponding author.

## Ethics statement

Ethical review and approval was not required for the study on human participants in accordance with the local legislation and institutional requirements. Written informed consent from the [patients/participants OR patients/participants legal guardian/next of kin] was not required to participate in this study in accordance with the national legislation and the institutional requirements.

## Author contributions

XZ: Conceptualization, Formal analysis, Writing – original draft, Writing – review & editing. JB: Conceptualization, Formal analysis, Writing – original draft, Writing – review & editing. MX: Conceptualization, Formal analysis, Investigation, Methodology, Writing – original draft, Writing – review & editing. JS: Conceptualization, Writing – review & editing. HX: Conceptualization, Investigation, Methodology, Writing – review & editing, Project administration.
