# Supplementary material for: Attitudes of Chinese residents toward sugar-sweetened beverage tax and their willingness to pay: a cross-sectional survey
Source: Front Nutr. 2023 Oct 25;10:1268436. doi: 10.3389/fnut.2023.1268436 (PMC10634395; doi:10.3389/fnut.2023.1268436)
Supplement: Supplementary file 1 [file Data_Sheet_1.docx]

Supplementary Material

**Table S1. Descriptive statistical analysis of model variables under different thresholds for Chinese adults according sociodemographic characteristics and nutritional status, 2021 (n=881).**

| **Variable** | **Variable description** | **Mean ± SD** | **Minimal values** | **Maximum values** |
| --- | --- | --- | --- | --- |
| Residents' attitude toward SSB tax | 1=Strongly disagree; 2=Somewhat disagree; 3=Neutral; 4=Somewhat agree; 5=Strongly agree | 3.43±1.125 | 1 | 5 |
| SSB expenses | Residents' monthly expenditure on the consumption of SSB (RMB) | 44.83±45.285 | 0 | 300 |
| Sex | 1=Male; 0=Female | 0.39±0.488 | 0 | 1 |
| Age | Age of residents on surveyed day | 27.36±7.001 | 16 | 66 |
| Household income level (Yuan per capita) | The total annual income of the resident family/Total family population (including the elderly, children, and Supported college students) | 10.87±0.834 | 6.9 | 12.6 |
| Education level | 1=High School and below; 2=Specialized;3=Undergraduate; 4=Master's and above | 4.96±0.661 | 1 | 4 |
| Residence | 1=Urban;0=Rural | 0.89±0.313 | 0 | 1 |
| Student | 1=Yes; 0=No | 0.43±0.495 | 0 | 1 |
| Is the profession related to food nutrition | 1=Yes; 0=No | 0.13±0.337 | 0 | 1 |
| BMI | 1=Underweight; 2=Healthy weight; 3=Overweight; 4=Obese | 2.06±0.672 | 1 | 4 |
| Physical exercises frequency | 0=No exercise; 1=<1 time per week; 2=1-2 times per week; 3＞=3 times per week | 1.95±0.882 | 0 | 3 |
| Self-assessed health status | 1=Poor; 2=Fair; 3=Good; 4=Very Good; 5=Excellent | 3.80±0.717 | 2 | 5 |
| Whether there is the intention or behavior to lose weight in the recent year | 0=Neither; 1=Intended, not tried; 2=Trying | 1.01±0.844 | 0 | 2 |
| Attitude toward taxing SSB | 1=Strongly disagree; 2=Somewhat disagree; 3=Neutral; 4=Somewhat agree; 5=Strongly agree | 3.43±1.125 | 1 | 5 |
| Attitudes toward regular consumption of SSB | 1=No impact；2=Low impact；3=Moderate impact；4=High impact；5=Higher impact | 4.08±0.799 | 1 | 5 |
| Are there children in the family who consume SSB regularly | 1=Yes; 0=No | 0.33±0.472 | 0 | 1 |
| Are there any adults in the household who consume SSB regularly | 1=Yes; 0=No | 0.48±0.500 | 0 | 1 |
| The time to the nearest sugary drink outlet | 1=Less than 3 minutes; 2=3-5 minutes; 3=5-10 minutes; 4=10-15 minutes; 5=More than 15 minutes | 2.26±0.930 | 1 | 5 |

**Table S2. Results of regression analysis of factors influencing Chinese adults’ attitudes toward SSB tax according sociodemographic characteristics and nutritional status, 2021 (n=881).**

| **Variable** |  | **W=1** | **W=2** | **W=3** | **W=4** |
| --- | --- | --- | --- | --- | --- |
| SSB expenses | Coefficient | 0.004 | -0.002 | -0.002 | -0.009 |
|  |  | (0.016) | (0.006) | (0.005) | (0.006) |
| Expenditures on SSB in quadratic terms | Coefficient | -0.000 | 0.000 | 0.000 | 0.000 |
|  |  | (0.000) | (0.000) | (0.000) | (0.000) |
| Perception of SSB | Coefficient | 0.373 | 0.543** | 0.705** | 1.530** |
|  |  | (0.236) | (0.126) | (0.119) | (0.226) |
| Are there children in the family who regularly consume the SSB | Coefficient | 0.454 | 0.411* | 0.425* | 0.308 |
|  |  | (0.434) | (0.206) | (0.170) | (0.229) |
| Are there any adults in the household who regularly consume the SSB | Coefficient | -0.403 | -0.558** | -0.472** | 0.007 |
|  |  | (0.493) | (0.197) | (0.174) | (0.226) |
| Distance to the nearest SSB outlet | Coefficient | 0.096 | 0.186 | 0.190* | -0.094 |
|  |  | (0.221) | (0.105) | (0.090) | (0.114) |
| Physical exercises frequency | Coefficient | 0.180 | 0.331** | 0.430** | 0.347* |
|  |  | (0.192) | (0.119) | (0.104) | (0.151) |
| Self-assessed health status | Coefficient | 0.291 | 0.295* | 0.304* | 0.438** |
|  |  | (0.310) | (0.142) | (0.119) | (0.170) |
| Whether there is the intention or behavior to lose weight in the recent year | Coefficient | 0.542* | 0.408** | 0.091 | 0.186 |
|  |  | (0.242) | (0.123) | (0.097) | (0.126) |
| Sex | Coefficient | -0.207 | -0.072 | -0.169 | 0.298 |
|  |  | (0.409) | (0.221) | (0.181) | (0.242) |
| Age | Coefficient | -0.048 | 0.047* | 0.045** | 0.025 |
|  |  | (0.040) | (0.019) | (0.015) | (0.015) |
| BMI | Coefficient | -0.005 | -0.005 | 0.020 | -0.021 |
|  |  | (0.011) | (0.024) | (0.022) | (0.030) |
| Educational or current qualifications | Coefficient | 0.037 | -0.076 | 0.170 | -0.204 |
|  |  | (0.311) | (0.170) | (0.130) | (0.151) |
| Logarithm of annual household income per capita | Coefficient | -0.274 | -0.033 | 0.017 | 0.307 |
|  |  | (0.256) | (0.115) | (0.103) | (0.167) |
| Residence | Coefficient | -0.116 | 0.325 | -0.299 | -0.264 |
|  |  | (0.688) | (0.266) | (0.230) | (0.336) |
| Are you a student | Coefficient | -0.506 | 0.173 | -0.002 | -0.116 |
|  |  | (0.439) | (0.228) | (0.200) | (0.268) |
| Is the profession related to food nutrition? | Coefficient | -0.019 | -0.146 | -0.107 | 0.055 |
|  |  | (0.620) | (0.285) | (0.229) | (0.316) |
| Area |  | Control | Control | Control | Control |
| Constant term |  | 3.451 | -4.645** | -7.146** | -13.257** |
|  |  | (2.935) | (1.650) | (1.420) | (2.204) |
| Observations |  | 850 | 850 | 850 | 850 |
|  |  | Wald $\chi^{2}$= 294.86 Pseudo $R^{2}=$0.1189 | | | |

Note: *, ** indicate significant at 5%, and 1% levels, respectively. Robust standard errors are presented in parentheses. Parameter estimates for W = 1 indicate that W = 1 is compared with W = 2, 3, 4, and 5; parameter estimates for W = 2 indicate that W = 1 and 2 are compared with W = 3, 4, and 5; parameter estimates for W = 3 indicate that W = 1, 2, and 3 are compared with W = 4 and 5; and parameter estimates for W = 4 indicate that W = 1, 2, 3, and 4 are compared with W = 5.

BMI, body mass index; SSB, sugar-sweetened beverage; W, the dependent variable (attitudes of residents toward sugar-sweetened beverage tax).
